# Ceftolozane/Tazobactam for Treating Children With Exacerbations of Cystic Fibrosis Due to *Pseudomonas aeruginosa*: A Review of Available Data

**DOI:** 10.3389/fped.2020.00173

**Published:** 2020-05-05

**Authors:** Silvia Garazzino, Elena Altieri, Erika Silvestro, Giulia Pruccoli, Carlo Scolfaro, Elisabetta Bignamini

**Affiliations:** ^1^Department of Child Pathology and Treatment “Regina Margherita”, Unit of Infectious Diseases, University of Turin, Città della Salute e della Scienza di Torino, Turin, Italy; ^2^Postgraduate School of Pediatrics, University of Turin, Turin, Italy; ^3^Division of Pulmonology, Pediatric Cystic Fibrosis Centre, Città della Salute e della Scienza di Torino, Turin, Italy

**Keywords:** Ceftolozane/tazobactam (C/T), cystic fibrosis—CF, children, pulmonary exacerbation, *Pseudomonas aeruginosa*

## Abstract

Ceftolozane-tazobactam is a novel fifth-generation cephalosporin/β-lactamase inhibitor combination recently approved for treatment of both complicated intra-abdominal and urinary tract infections in adults. Considering its potent bactericidal activity against *Pseudomonas aeruginosa*, it might represent an important option also for treating children with exacerbations of cystic fibrosis due to *Pseudomonas aeruginosa* when other alternative treatments have been exhausted. We hereby review available data on the use of ceftolozane-tazobactam in children, focusing on cystic fibrosis.

## Introduction

*Pseudomonas aeruginosa* is the leading cause of acute respiratory exacerbations in patients affected by cystic fibrosis (CF), both adults and children. Chronic infections are related with a progressive decline in pulmonary function; consequently, aggressive antimicrobial treatment of exacerbations is essential to improve expectancy and quality of life, especially in patients awaiting lung transplantation (1). However, with the widespread of multidrug-resistant (MDR) strains, the choice of an appropriate antimicrobial treatment is becoming more difficult, especially in the pediatric setting, where the new available drugs are often off-label in terms of age, indication, and dosage.

Ceftolozane-tazobactam (C/T) is a novel fifth-generation cephalosporin/β-lactamase inhibitor combination with activity against MDR Gram-negative bacilli that has been approved by the Food and Drug Administration and the European Medicines Agency for treatment of complicated intra-abdominal infections (cIAIs), complicated urinary tract infections (cUTIs), pyelonephritis, and hospital-acquired pneumonia (HAP) in adult patients. Ceftolozane-tazobactam has been successfully used for treating acute pulmonary CF exacerbations in adults, although not licensed for such indication (2, 3). Because of its spectrum of activity, C/T might represent an important option also for CF children with *P. aeruginosa* infections when other alternative treatments have been exhausted.

To date, the clinical experience with the use of C/T in children is extremely limited, dosing data are scanty, and only a few of the agreed Pediatric Investigation Plans have been completed (4).

We hereby review the available data on the use of C/T in children, focusing on CF patients.

## Mechanism of Action and Microbiologic Data

Ceftolozane belongs to the class of cephalosporins and is structurally similar to ceftazidime, but it has a modified 3-position pyrazole side chain that increases its activity against *P. aeruginosa*. Its potent bactericidal activity relies upon inhibition of bacterial cell-wall synthesis through inhibition of penicillin-binding proteins (PBPs); compared to ceftazidime, ceftolozane displays a higher affinity for the *P. aeruginosa* PBPs 1b,1c, and 3 and has an increased stability to AmpC β-mediated resistance, which is common among *P. aeruginosa* strains (5, 6).

Tazobactam is a potent, irreversible inhibitor of most class A and some class C β-lactamases, which broadens ceftolozane's activity to most extended-spectrum β-lactamase (ESBL)–producing Gram-negative bacteria and confers some anaerobic activity (7, 8).

The fixed dose combination currently available with a 2:1 ratio of ceftolozane and tazobactam comes from studies comparing the efficacy of different ratio combinations (2:1, 4:1, and 8:1), in which the 2:1 appeared the most potent (9).

The strong activity of ceftolozane alone and of C/T against β-lactam–resistant (including meropenem-resistant) *P. aeruginosa* has been widely documented in non-CF strains isolated from adults and children, showing a superiority of C/T in comparison to ceftazidime-avibactam (CZA) in terms of susceptibility *in vitro* (10–15). In the study by Humphries et al. (16), more than one-third (36%) of CZA-resistant isolates resulted C/T susceptible, whereas only 9% C/T-resistant isolates were still CZA susceptible.

In an Italian nationwide survey on *P. aeruginosa* isolated from invasive infections, C/T appeared the most active molecule, retaining activity against 90.9% of isolates, followed by amikacin (88.0% susceptibility) and colistin (84.7% susceptibility) (17).

Also according to a global surveillance program including isolates from inpatients in four continents, including children, C/T displays the highest activity against *P. aeruginosa* among the currently available drugs except for colistin (12, 13).

Resistance to C/T may be driven by different mechanisms, such as OprD membrane protein loss or downregulation, overexpression of AmpC β-lactamase, or overexpression of efflux pumps (17–21).

It must be considered that the characteristics of bacteria isolated from CF patients differ from those found in the general population. Indeed, in the respiratory tract of CF patients, where bacterial eradication is hardly attainable once colonization has been established, mucoid, and hypermutable *P. aeruginosa* strains are highly relevant. Several studies on *P. aeruginosa* isolates from patients with CF are available, showing a potent, concentration-independent bactericidal activity of C/T also on mucoid and resistant isolates, although percentages of susceptibility to C/T are on average lower than those reported in non-CF strains (10, 22–24).

No relevant activity of C/T against *Burkholderia cepacia* has been reported (23).

In one of the first studies on MDR *P. aeruginosa* isolates from CF adults, ceftolozane resulted the most active among tested drugs, although up to 36% of the isolates had a minimal inhibitory concentration (MIC) of >8 μg/mL (23).

Gramegna et al. (25) analyzed a panel of 120 *P. aeruginosa* isolates from CF adult patients, including 42.5% of MDR/extensively drug-resistant (XDR) strains and 2.5% of pan–drug-resistant (PDR) strains. Within the β-lactams tested, C/T had the lowest percentage of *in vitro* antimicrobial resistance against *P. aeruginosa*: 84.2% isolates were susceptible to C/T, and only two were highly resistant (MIC >256 mg/mL). The MIC_50_ and MIC_90_ values for C/T against *P. aeruginosa* were 1.5 and 8 mg/mL, respectively. Ceftolozane-tazobactam appeared effective in most of the meropenem-resistant *P. aeruginosa* strains (25).

In a smaller sample of XDR and PDR *P. aeruginosa* also collected from adult CF patients, C/T showed the highest percentage of *in vitro* susceptibility (30 vs. <10% of other antibiotics tested). Considering only XDR isolates, susceptibility to C/T was reported in 43% of cases. None of the nine PDR strains were susceptible to C/T, although two had intermediate MICs (26).

In a more recent study, the *in vitro* activity of C/T against *P. aeruginosa* in CF patients was comparable to colistin (susceptibility rate 85.1 vs. 89.4%) but significantly higher than other antimicrobials (27). Ceftolozane-tazobactam was active against 70, 58.1, and 100% of MDR, XDR, and PDR strains, respectively. No differences in C/T activity were observed between isolates from children and adult patients, except for XDR ones that resulted significantly more susceptible in older patients. Activity of C/T toward mucoid isolates was inferior to colistin (82.9 vs. 97.6%) but higher compared with other antibiotics.

To our knowledge, only two further studies evaluated the *in vitro* activity of C/T on *P. aeruginosa* strains that were isolated from children affected by Kuti et al. (28) tested C/T on 50 non-duplicate MDR *P. aeruginosa* strains, of which 48% had mucoid phenotypes: C/T appeared the most active antimicrobial, with an 86% susceptibility that was not influenced by treatment; 10% of isolates resulted intermediate, and 4% were resistant to C/T. Time-kill analyses of C/T alone and in combination with tobramycin or amikacin against five *P. aeruginosa* isolates from four pediatric CF patients demonstrated that concentrations of at least 8 × C/T MIC are required for an adequate killing (29). The synergistic activity of aminoglycoside combination, when the isolate was C/T susceptible, was more pronounced with amikacin than with tobramycin (29).

## C/T Clinical Data in Adult Patients With *P. aeruginosa* Infection

The first data on efficacy and safety of C/T in the adult population came from two phase 3 randomized clinical trials (RCTs), called, respectively, ASPECT-cIAI and ASPECT-cUTI, in which C/T non-inferiority with respect to meropenem and levofloxacin allowed the formal approval of the investigational drug for the treatment of cIAIs and cUTIs (30, 31). A third RCT, ASPECT-NP, comparing C/T vs. meropenem for hospital-acquired bacterial pneumonia (HABP) or ventilator-associated bacterial pneumonia (VABP), was recently concluded and lead to the inclusion of severe respiratory infections within the labeled indications (32). Such registration trials included few patients infected with MDR bacteria, although this is the actual field where C/T addresses an unmet medical need.

In real life, C/T has been used for treating a broad spectrum of severe infections due to susceptible pathogens, including ESBL-producing Enterobacteriaceae (according to a carbapenem-sparing strategy) and *P. aeruginosa* (33). However, observational studies reporting the use of C/T for treating MDR *P. aeruginosa* in adults consist of case series and case reports.

In a retrospective study involving 22 Italian hospitals, Bassetti et al. (34) described a wide adult population (101 patients) treated with C/T for different *P. aeruginosa* infections, including nosocomial pneumonia (31.7%), acute bacterial skin and skin-structure infection (20.8%), cUTI (13.9%), cIAI (12.9%), bone infection (8.9%), and primary bacteremia (5.9%). Barely a half (50.5%) of *P. aeruginosa* isolates were XDR, with the majority resistant to at least one carbapenem. Overall clinical success was 83.2%, but significantly lower rates were reported in patients with sepsis or under continuous renal replacement therapy. Ceftolozane-tazobactam was mainly used as second-line or later with a median duration of 14 days. In more than one-third of patients, it was concomitantly administered with other antipseudomonal agents, such as aminoglycosides, colistin, and carbapenems.

A more recent study analyzed 259 adults treated with C/T for MDR Gram-negative infections; *P. aeruginosa* was the causative agent in the majority (91.1%) of patients, and the most common infection source was the respiratory tract (35). Clinical failure was reported in 37.6% of cases and was independently related to hospital-acquired infection and higher Acute Physiology and Chronic Health Evaluation II score. Combination intravenous (IV) antibiotic therapy was used in 24.7% of patients, most commonly an aminoglycoside. Adjuvant therapy with inhalation tobramycin or colistin was administered in 29.4% of patients with a respiratory tract infection (RTI).

In general, the use of combination therapy for the management of infections caused by *P. aeruginosa* has been explored *in vitro* and *in vivo* with the objective of investigating the potential synergistic or additive effects of certain combinations of drugs with different mechanisms of action (36). Available data suggest that combination therapy may be beneficial for treating severe infections sustained by MDR Gram-negative bacteria in high-risk patients, such as those affected by CF, whereas monotherapy may be enough for lower-risk patients (37). Combination therapy may be beneficial also to prevent the development of antimicrobial resistance (38). However, this is counterbalanced by the additional risk of toxicity (mainly kidney insufficiency) and by the poor pulmonary penetration and diminished antibacterial activity of aminoglycosides in the acidic pneumonic airways (35).

## C/T Dosage in CF Adult Patients

The approved C/T dosage in adults is 1.5 g IV every 8 h (q8h) for cUTIs and cIAIs, whereas a higher dose comprising 2 g of ceftolozane and 1 g of tazobactam q8h is recommended for treating either HABP or VABP (39, 40).

Before formal approval, in clinical practice the high-dose regimen C/T (9 g daily) had been already used in a large amount of adult patients with MDR Gram-negative bacterial infections, mostly with RTIs (35).

In patients with lower RTIs caused by MDR and XDR *P. aeruginosa*, higher MICs (>2 mg/L) were found to be associated with lower efficacy of C/T and higher 30-day mortality (*P* = 0.045) independently from the dosage used (41). However, it has been demonstrated that in patients receiving continuous infusion of C/T the pharmacokinetic/pharmacodynamic (PK/PD) target is achieved even for strains with MICs equal to 8 mg/L (42).

The 3 g q8h RTIs dosage has been safely and successfully used in CF adults with pulmonary exacerbations (2, 43). Patients with CF have been reported to have altered PKs, in terms of larger volume of distribution, increased total body clearance of β-lactams, lower exposure, and shorter elimination half time; therefore, maximal dosages are often required to achieve adequate plasma and epithelial lining fluid concentrations of antimicrobials (44). Nevertheless, in 20 CF patients, C/T clearance resulted similar to what was observed in non-CF adults, although the volume of the central compartment was lower (43). The results of the Monte Carlo simulation lead the authors to recommend the use of 3 g q8h also for treating pulmonary CF exacerbations in adults: a >90% probability of the PD target attainment exposure of 60% free time above the MIC (*f* T> MIC) was achieved at MICs up to 4 and 8 μg/mL with the 1.5- and 3-g dosage, respectively (43). At present, whether C/T higher dosages, extended infusion regimens, or reduced dosing intervals are required in CF adults with pulmonary exacerbations has not been sorted out.

A continuous infusion regimen of C/T, associated to therapeutic drug monitoring to assess adequacy of exposure, was successful in a CF adult patient with augmented renal clearance (45).

## Pharmacokinetic and Clinical Data in the Pediatric Population

Pharmacokinetic and safety of C/T were evaluated in a phase I trial enrolling 37 neonates and children up to 17 years of age with Gram-negative infections (46–48). Single IV age-based doses, ranging from 18 to 30 mg/kg up to 1.5 g of ceftolozane with a fixed 2:1 ratio of C/T, were well-tolerated and resulted in ceftolozane PK parameters generally comparable among various age groups of children older than 3 months. Patients <3 months of age had a lower clearance and a slightly higher volume of distribution of C/T compared to older children, consistent with an immature renal function in this cohort and given that both products are cleared by the kidney.

Of note, the interim analysis led to the increase of C/T dosage to 30/15 mg/kg in children aged >3 months to <7 years and to 20/10 mg/kg in neonates and infants <3 months of age.

Larson et al. (49) developed a population PK (popPK) model of C/T integrating PK data from 12 adult clinical trials (healthy volunteers, volunteers with renal impairment, and patients with cUTIs o cIAIs) and the above mentioned phase I pediatric study. The popPK analysis showed that a two-compartment linear model with first-order elimination well-describes the concentration vs. time profile of C/T, that renal function significantly affects C/T clearance, and that body weight influences both C/T clearance and volume of distribution (49). The popPK model guided dose selection for two ongoing phase 2 trials evaluating C/T for the treatment of cUTIs and cIAIs in children: 1.5 g q8h over a 1-h infusion for children 12 to <18 years old and 20/10 mg/kg q8h for children <12 years old (50, 51).

In the pediatric phase I trial, several children with CF were enrolled; an unpublished analysis of C/T PK using model-derived parameters showed no relevant differences between children with or without CF (52).

To date, five pediatric patients treated with C/T for MDR or XDR *P. aeruginosa* infection have been described; each child had serious underlying comorbidities (Table 1).

**Table 1:** Characteristics and outcomes of five children with *Pseudomonas aeruginosa* infection treated with Ceftolozane/tazobactam.

**Author (year)**	**Age (years)**	**Sex**	**Type of infection**	**Concomitant disease(s)**	**C/T MIC**	**C/T treatment (daily dosage, duration)**	**Ceftolozane C_**min**_**	**C/T-related AEs**	**Concomitant antimicrobials**	**Outcome**
Aitken et al. (53)	9	M	*P. aeruginosa* BSI	Acute myeloid leukemia	6 μg/mL	50 mg/kg C every 8 h over a 3-h infusion (first treatment), 3 weeks)	5.2 μg/mL	None	Tobramycin, ciprofloxacin	Recovered after a first relapse
					8 μg/mL	40 mg/kg C every 6 h over a 3-h infusion (second treatment), 3 weeks	18.1 μg/mL			
Zikri and Masri (54)	14	F	MDR *P. aeruginosa* pneumonia	Combined immunodeficiency syndrome	3 μg/mL	1 g (C) + 0,5 g (T) every 8 h, corresponding to 44/22 mg/kg (C/T) every 8 h, NA	NA	None	Amikacin, colistin	Recovered
Ang et al. (55)	14	F	*P. aeruginosa* pulmonary exacerbation	Cystic fibrosis	Mucoid strain 0.5 μg/mL; non-m ucoid strain MIC 1 μg/mL	1 g (C) + 0,5 g (T) every 8 h, corresponding to 31.25 mg/kg (C) every 8 h, 14 days	1.2 μg/mL	None	Ciprofloxacin	Recovered
Martín-Cazaña et al. (56)	5	M	*P. aeruginosa* endocarditis	Complex congenital heart disease	2 μg/mL	50 mg/kg (C) every 8 h over 3-h infusion, 45 days	2.6 ng/mL	None	Tobramycin	Recovered
Dinh et al. (57)	3	M	XDR *P. aeruginosa* vascular graft infection	Liver transplant	NA	1.5 g (C) + 0.75 g (T)/day, 57 days	NA	*Clostridium difficile* infection	Colistin	Failure

*C, ceftolozane; T, tazobactam; AEs, adverse events; M, male; F, female; BSI, bloodstream infection; MDR, multidrug resistant; NA, not available*.

A therapeutic failure was recorded under C/T treatment (57 days) in a liver transplant recipient with XDR *P. aeruginosa* vascular graft infection (57). On the other hand, C/T was successfully used to treat bilateral pneumonia with septic shock in a girl with combined immunodeficiency syndrome, a bloodstream infection in a 9-year-old boy with relapsed/refractory acute myeloid leukemia, and an endocarditis in a child with congenital heart defect (53, 54, 56). In all these patients, C/T was well-tolerated, even for prolonged treatments. Whenever performed, PK analysis demonstrated appropriate plasma levels of ceftolozane with respect to the MIC of the isolate.

One single case regarding the use of C/T in pediatric patients with CF was retrieved in the literature (55). Ang et al. (55) described a 14-year-old girl with CF and MDR *P. aeruginosa* pulmonary exacerbations; notably after desensitization due to β-lactam allergy, she was treated with C/T at a dosage of 1.5 g q8h over a 1-h infusion, which was equivalent, in milligrams per kilograms, to the adult 3 g q8h HAP dosage. A PK study was performed at steady state, and PK parameters of C/T were then modeled to simulate a variety of C/T dosing regimens, such as 1.5 g q8h, 1.5 g q6h, and 3 g q8h, each given as 1- or 3-h infusion. All regimens exceeded the PD target *f* T>MIC of 40%, but authors postulated that the use of higher dosages, more frequent administrations, and/or prolonged infusion of C/T might be necessary to guarantee PD optimization when *P. aeruginosa* MIC for C/T is 4 to 8 μg/mL.

## Conclusions

To date, C/T appears one of the most active drugs against *P. aeruginosa*, representing an important new therapeutic option for difficult-to-treat infections due to MDR strains, also outside approved indications (4).

The favorable safety profile of tazobactam in children is well-known because of the consolidate use of the association with piperacillin in hospitalized pediatric patients. Ceftolozane appears to be well-tolerated in adults even at high dosages; according to phase I clinical trials results in children, that is expected to occur also in the pediatric population where other IV cephalosporins are routinely and safely used. All these properties make C/T a possible therapeutic strategy to treat children with CF pulmonary exacerbations due to MDR *P. aeruginosa* (58, 59). Awaiting for more robust data in the pediatric population and inferring from the real-life experience in adults, we suggest using C/T in combination with other antimicrobial agents, such as aminoglycosides or colistin, to treat serious infections, in order to prevent resistance selection and to obtain an eventual synergistic effect.

However, a few considerations are due. First, it is debated whether susceptibility patterns should guide or not the selection of antibiotics to treat CF exacerbations, as some data show that *in vitro P. aeruginosa* susceptibility patterns do not predict clinical response (60). Secondly, limiting the use of new antimicrobials is a crucial element of the antimicrobial stewardship program; therefore, C/T prescription should be prerogative of infectious diseases specialists and limited to selected cases where other therapeutic options are unfeasible, aiming at preventing drug overuse and rapid selection of microbial resistance. Finally, the proper C/T dosing regimen has not yet been defined in CF patients and especially in children. Given the available data from clinical studies and from popPK models, it may be inferred that in CF children with pulmonary infections and a normal renal function a ceftolozane dose of at least 30–40 mg/kg q8h (according to age) is required. However, given that CF patients may have altered drugs' pharmacokinetics, additional strategies, such as prolonged infusions or q6h dosing, may be necessary to guarantee adequate drug exposure, especially if the MIC of the causative organism is unfavorable. Additional clinical and PK studies on C/T use in children with CF are warranted before any definite recommendation could be made.

## Author Contributions

All authors significantly contributed to the present manuscript. SG designed the study, reviewed the literature and wrote the manuscript. EA, ES, GP, CS, and EB reviewed the literature, corrected the manuscript and edited English style.

## Conflict of Interest

The authors declare that the research was conducted in the absence of any commercial or financial relationships that could be construed as a potential conflict of interest.

## Abbreviations

C/T: ceftolozane-tazobactam
CF: cystic fibrosis
cIAI: complicated intra-abdominal infection
cUTI: complicated urinary tract infection
CZA: ceftazidime-avibactam
ESBL: extended-spectrum β-lactamase
fT> MIC: free time above the MIC
HABP: hospital-acquired bacterial pneumonia
HAP: hospital-acquired pneumonia
IV: intravenous
MIC: minimal inhibitory concentration
MDR: multidrug-resistant
PBPs: penicillin-binding proteins
PD: pharmacodynamic
PDR: pan-drug resistant
PK: pharmacokinetic
PopPK: population PK
Q8h: every 8 h (quaque 8 h)
RTI: respiratory tract infection
VABP: ventilator-associated bacterial pneumonia
XDR: extensively drug-resistant.
